# Social Determinants of Disparities in Mortality Outcomes in Congenital Heart Disease: A Systematic Review and Meta-Analysis

**DOI:** 10.3389/fcvm.2022.829902

**Published:** 2022-03-15

**Authors:** Richard Tran, Rebecca Forman, Elias Mossialos, Khurram Nasir, Aparna Kulkarni

**Affiliations:** ^1^Department of Health Policy, London School of Economics and Political Science, London, United Kingdom; ^2^Department of Cardiology, Houston Methodist DeBakey Heart and Vascular Institute, Houston Methodist Hospital, Houston, TX, United States; ^3^Cohen Children’s Medical Center, Donald and Barbara Zucker School of Medicine, New Hyde Park, NY, United States

**Keywords:** congenital heart disease, death, social determinants of health, health disparities, health inequalities

## Abstract

**Background:**

Social determinants of health (SDoH) affect congenital heart disease (CHD) mortality across all forms and age groups. We sought to evaluate risk of mortality from specific SDoH stratified across CHD to guide interventions to alleviate this risk.

**Methods:**

We searched electronic databases between January 1980 and June 2019 and included studies that evaluated occurrence of CHD deaths and SDoH in English articles. Meta-analysis was performed if SDoH data were available in >3 studies. We included race/ethnicity, deprivation, insurance status, maternal age, maternal education, single/multiple pregnancy, hospital volume, and geographic location of patients as SDoH. Data were pooled using random-effects model and outcome was reported as odds ratio (OR) with 95% confidence interval (CI).

**Results:**

Of 17,716 citations reviewed, 65 met inclusion criteria. Sixty-three were observational retrospective studies and two prospective. Of 546,981 patients, 34,080 died. Black patients with non-critical CHD in the first year of life (Odds Ratio 1.62 [95% confidence interval 1.47–1.79], I^2^ = 7.1%), with critical CHD as neonates (OR 1.27 [CI 1.05-1.55], I^2^ = 0%) and in the first year (OR 1.68, [1.45-1.95], I^2^ = 0.3%) had increased mortality. Deprived patients, multiple pregnancies, patients born to mothers <18 years and with education <12 years, and patients on public insurance with critical CHD have greater likelihood of death after the neonatal period.

**Conclusion:**

This systematic review and meta-analysis found that Black patients with CHD are particularly vulnerable for mortality. Numerous SDoH that affect mortality were identified for specific time points in CHD course that may guide interventions, future research and policy.

**Systematic Review Registration:**

[https://www.crd.york.ac.uk/PROSPERO/display_record.php?ID=CRD42019139466&ID=CRD42019139466], identifier [CRD42019139466].

## Introduction

Race and ethnicity are known to be associated with varied outcomes in patients with congenital heart disease (CHD). A recent study of CHD related United States national deaths by Lopez et al. summarized that from 1999 to 2017 the age-adjusted CHD mortality had improved, but Black patients with CHD remained at higher risk for death than White patients (1). Delving deeper into the upstream factors that contribute to health disparities within these patient communities is key to implementing actionable health policies and interventions to improve outcomes (2, 3). These factors, known as the social determinants of health (SDoH)—“the conditions in which people are born, grow, live, work, and age” (4)—lead to unequal health outcomes in younger people that are then perpetuated through the life course (5–9). It is known that racial/ethnic minority groups are more likely to experience structural barriers to access of healthcare, living in safe physical environments, attaining high quality education, and gaining stable employment that may result in a cumulative social disadvantage. Present data have evaluated SDoH in select populations with CHD in limited and distinct acute care or outpatient settings. This systematic review and meta-analysis (SR-MA) was designed to compile and summarize all the existing evidence in SDoH and their effects on mortality across the entire spectra of CHD and across all age groups, so factors that affect outcomes at various stages of disease progression may be better understood, and thereby intervened upon.

## Materials and Methods

The protocol for this SR-MA was finalized *a priori* and registered with PROPSERO (Registration no. CRD42019139466). Specific elements related to Population, Intervention, Comparison, Outcome, and Setting (PICOS) that comprised the PICOS question were as follows:

1. Population: All patients from birth to adult age with CHD
2. Intervention: None specified
3. Comparison: None
4. Outcome: Primary outcome of death
5. Setting: Inpatient and outpatient

### Study Search and Selection Criteria

A comprehensive literature search was conducted to identify studies published in the English language between January 1980 and June 2019 that reported on mortality and survival from CHD, separated by SDoH category. The database searches used a combination of Medical Subject Headings (MeSH) and keywords for social determinants of health, mortality and congenital heart disease in four electronic databases: OVID Medline, OVID Embase, Web of Science (Core Collection) and CINAHL (Supplementary Section 1). Iteration of reference lists from included articles was performed to identify studies that were not returned in the database search.

### Study Selection and Data Extraction

Title and abstract screening was performed using the Rayyan web application (10), followed by full text screening by two reviewers (RT and RF). Data were abstracted for individual study by the primary reviewer (RT) and checked for accuracy by the second reviewer (RF). Conflicts were resolved by consensus and discussion with a third reviewer (AK). Preferred Reporting Items for Systematic Reviews and Meta-analyses (PRISMA) guidelines were followed (11).

### Inclusion and Exclusion Criteria

Randomized and non-randomized clinical trials, observational and case-control studies on mortality and survival from CHD separated by SDoH group were included in the study. Case reports, case series with <10 patients, systematic/narrative reviews, abstracts only, editorials, letters to editors, commentaries, studies reporting prenatal outcomes and focused solely on gender, nutrition, and patent ductus arteriosus were excluded.

### Data Abstraction

Data were abstracted for individual study by the primary reviewer (RT) and checked for accuracy by the second reviewer (RF).

### Study Definitions

Relevant social, economic, environmental, cultural, and behavioral SDoH were included in the analysis. We noted that standard and/or similar definitions of SDoH have not been used across all the included studies. However, in order to gain a comprehensive understanding of the SDoH and their impact on mortality in CHD, we chose to include and analyze studies providing data on most SDoH and categorized patients whenever possible and if needed within standard definitions available. For example, deprivation was classed in different studies either on the basis of household income or relative poverty/deprivation level. For this study, we analyzed patients by categorizing them as the most and least deprived. Another such example is for hospital status, based on case volume or teaching hospital. In this instance, we chose to keep the original study definitions of high volume or teaching hospital versus low volume or non-teaching hospital. Similarly, geographical barriers for access to care were explored by using a combined category of urban versus rural hospital or residence, and distance to hospital, without altering the original definitions used in the included studies. Details of definitions of all SDoH used in the included studies and their categorizations are provided in Supplementary Section 2.

### Statistical Analysis

Summary statistics were used to describe extracted data. The primary outcome of interest was mortality; no secondary outcomes were studied. Meta-analysis was carried out to determine a pooled estimate of the direction of effect and degree of association between a SDoH and CHD mortality only if three or more studies met the inclusion criteria. To facilitate comparison between patient groups, data were stratified by timing of mortality (inpatient mortality, neonatal mortality, mortality in the first year and long-term mortality after 1 year) and risk category. All ductal dependent CHD or those with significant operative intervention or postoperative care were classified as critical CHD (Table 1). When it was not possible to perform an analysis on a particular SDoH using studies from the same risk category or mortality timing group (due to lack of clarity within the studies), analyses were carried out to understand a possible overall effect of this SDoH on CHD mortality. If two or more studies from the same cohort or database and timeframe qualified, both studies were included in the overall results, but only the study with the greatest number of participants was included in the MA. If more than one CHD type from the same risk category was present in a study and it was not possible to combine data (for example, when patients had two or more coexistent forms of CHD), data for the CHD type that best facilitated comparison with other studies was extracted and used in MA.

**TABLE 1:** Risk categories used for systematic review and meta-analysis.

Risk category presented in or inferred from study	Risk category used in systematic review and meta-analysis
RACHS 1 or 2; STAT 1 or 2	Non-critical CHD
RACHS 3-6; STAT 3-5	Critical CHD

*RACHS, risk adjustment in congenital heart surgery score; STAT, the society of thoracic surgeons-european association for cardiothoracic surgery score.*

A random effects model was used to calculate odds ratios. The true effect size was assumed to vary between studies. The chi-square test for homogeneity was utilized to assess heterogeneity when combining studies with similar exposure and outcome measures. Statistical significance was defined at *p* <0.1 rather than *p* <0.05 level, given the low power of this test in a meta-analysis when there are few included studies or studies with small sample sizes (12). An I^2^ statistic was calculated to quantify heterogeneity in the MA. An I^2^ ≤ 30% was considered to have low heterogeneity, 30–50% moderate heterogeneity and >50% was likely to represent substantial or considerable heterogeneity (12). All analyses were performed using STATA Version 16.1, College Station, TX, United States.

### Risk of Bias Assessment

All included studies were assessed for risk of bias (ROB) by the primary reviewer (RT) and checked for accuracy by the secondary reviewer (RF). All studies were observational, non-randomized; therefore, the Cochrane Risk of Bias in Non-Randomized Studies (ROBINS-I) tool was used. ROB was classed as low, moderate, serious or critical (13). A funnel plot was generated for studies that included data on race/ethnicity (the variable with the largest number of included studies) to assess publication bias.

### Ethics

This project was judged by the Research Governance & Integrity Office at the London School of Hygiene and Tropical Medicine as exempt from ethical review when RT was affiliated with the institution in 2019. Individual patient health data were not accessed for this study.

## Results

**FIGURE 1:**
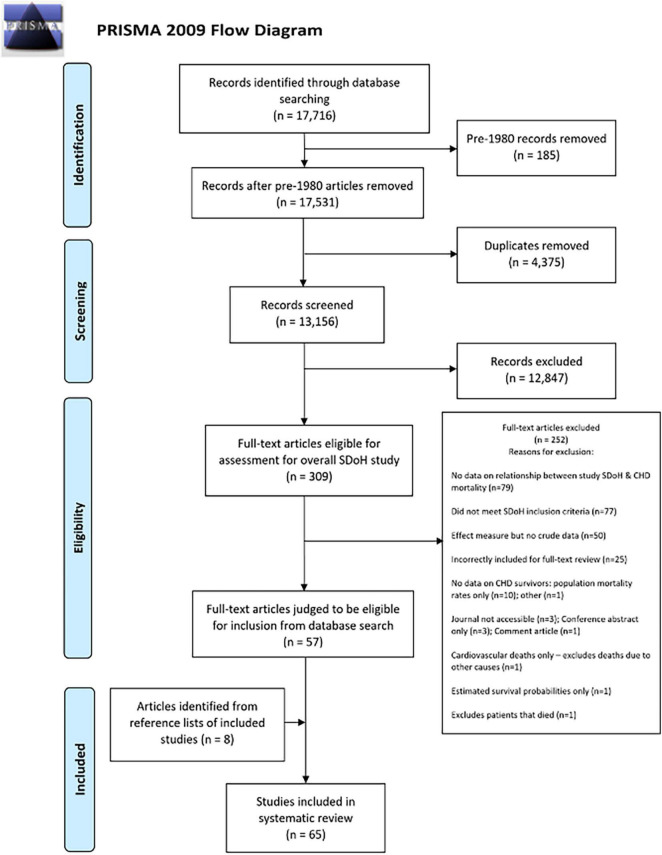
PRISMA flowchart showing inclusion/exclusion process for systematic review and meta-analysis.

A total of 17,716 citations met the search criteria, of which 13,156 studies underwent title and abstract screening. Full-text review was performed on 309 articles, and 57 met inclusion criteria. Eight additional studies were identified on iteration of references. Search results are presented in Figure 1 using the PRISMA flowchart.

### Study Characteristics

Of the 65 studies included in the review (2, 14–77), 63 were retrospective, observational studies; two were prospective. Data from regional, national, or international databases or registries was published in 53 studies. Forty-eight studies (74%) contained data exclusively from the United States. The basic characteristics of the 65 included studies are described in Supplementary Table 1.

Among the total 546,981 patients with CHD, 34,080 died. The age range of the patients ranged from 0 days to 91 years, and seven studies included data on adult CHD (ACHD) and/or followed patients up for more than 20 years (22, 45, 67, 69–72). Although adult data were described in the individual studies, sufficient SDoH variables were not available on these patients to allow for MA in the adult age group. Not all patients underwent surgical interventions, and thus unrepaired ACHD patients were included in the analysis [Verheught et al. (71)]. Mortality according to both SDoH and CHD risk categories could be determined in 40 studies. The number of studies that published data on each SDoH were as follows: race/ethnicity (*n* = 52), deprivation status (*n* = 16), insurance status (*n* = 15), maternal age (*n* = 12), maternal education (*n* = 9), single/multiple pregnancy (*n* = 5), urban/rural hospital/residence or distance to specialist care (*n* = 7), hospital case volume (*n* = 11), surgery at teaching/non-teaching hospital (*n* = 3), parental occupation (*n* = 2), receiving public assistance (*n* = 1), and number of adult care givers (*n* = 1). Only relevant positive and negative results are discussed due to the large number of variables analyzed.

### Synthesis of Pooled Results

#### Race and Ethnicity (Figures 2, 3)

**FIGURE 2:**
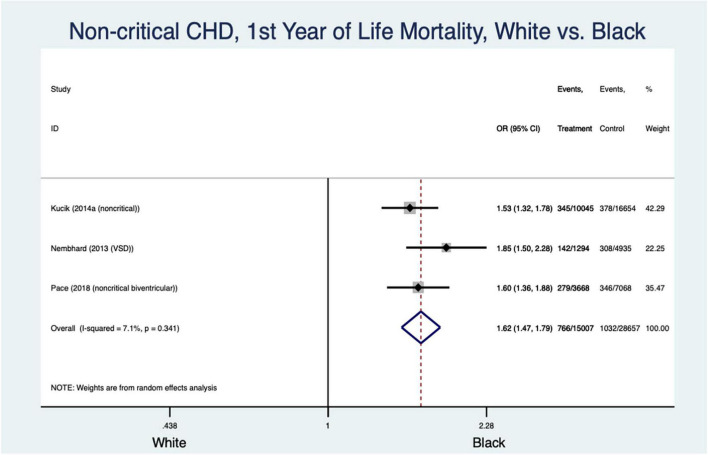
Forest plot demonstrating odds ratio for mortality in non-critical congenital heart disease in the first year of life based on White vs. Black race/ethnicity.

**FIGURE 3:**
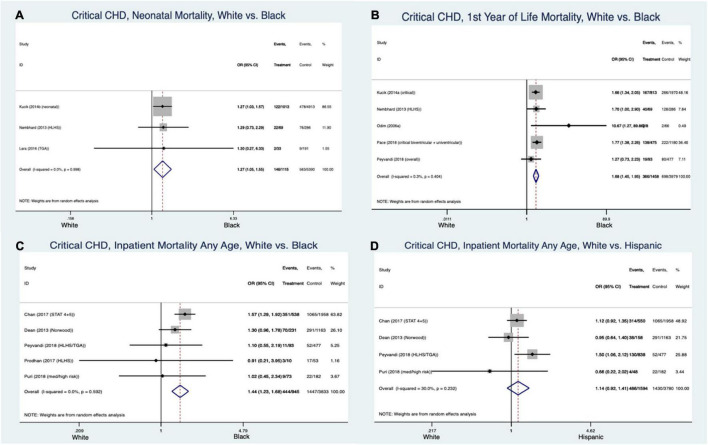
Forest plots demonstrating odds ratio for mortality in critical congenital heart disease at various stages in early life based on White vs. Black race/ethnicity **(A–C)** and White vs. Hispanic **(D)** race/ethnicity.

There was an increased odds of mortality in Black patients compared to Whites with non-critical CHD in the first year of life (odds ratio (OR) 1.62 [95% confidence interval 1.47–1.79], I^2^ = 7.1%) (Figure 2). This analysis could not be interpreted in the pooled MA of Hispanic patients with non-critical CHD compared to their White counterparts for the same age group due to significant heterogeneity in the data (OR 0.96 [CI 0.57–1.57], I^2^ = 95.7%). Black patients (Figure 3A) with critical CHD were more likely to die than White patients as neonates (OR 1.27 [CI 1.05–1.55], I^2^ = 0%) and in the first year of life (OR 1.68, [1.45–1.95], I^2^ = 0.3%) (Figure 3B). No such clear association was seen beyond the first year of life (OR 1.47 [CI 0.82–2.63], I^2^ = 81.3%). Overall inpatient mortality across all ages with critical CHD in Black patients remained higher than in White patients (OR 1.44 [CI 1.23–1.68], I^2^ = 0%) (Figure 3C). Conversely, Hispanic patients with critical CHD did not appear to have such clear trends for mortality at birth (OR 1.17 [CI 0.98–1.4], I^2^ = 13.7%) and data were too heterogenous at <1-year (OR 1.22 [CI 0.9–1.66], I^2^ = 72.3%) and >1-year of age (OR 0.94 [CI 0.58–1.53], I^2^ = 74.6%) to assign odds of mortality. Overall inpatient mortality across all ages (Figure 3D) did not show significant difference between Hispanic and White patients (OR 1.14 [CI 0.92–1.41], I^2^ = 30%).

#### Socioeconomic Factors (Figure 4)

**FIGURE 4:**
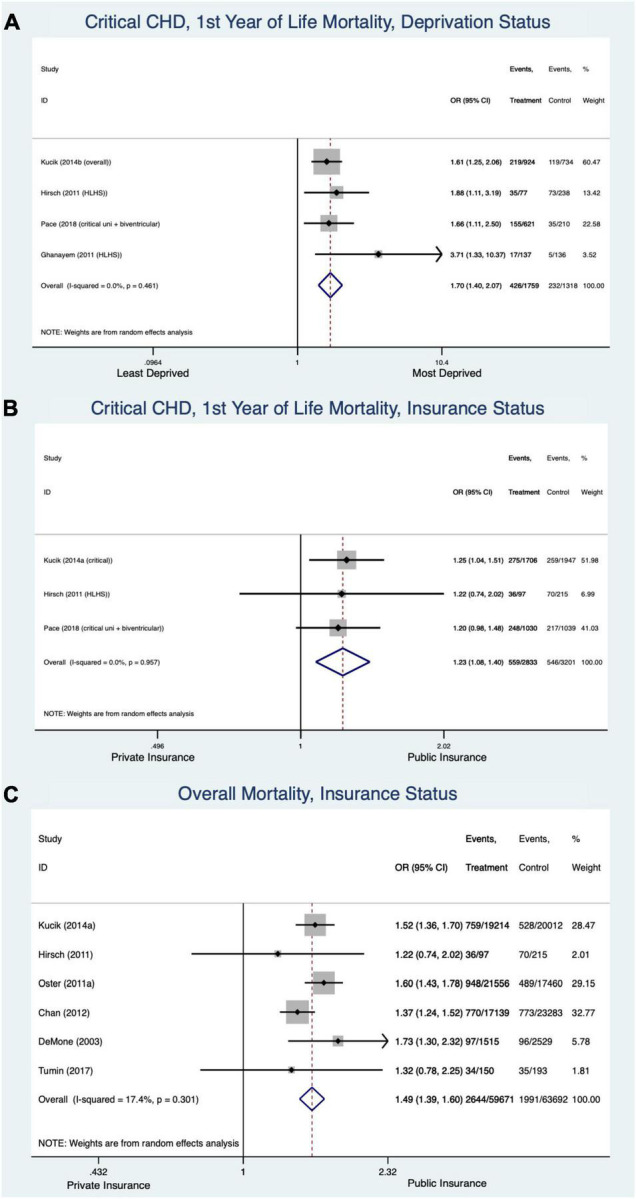
Forest plots demonstrating odds ratio for mortality in congenital heart disease based on socioeconomic factors, deprivation status in the first year of life **(A)**, insurance status in first year of life **(B)**, and insurance status across all ages and forms of congenital heart disease **(C)**.

The socio-economically deprived patients with critical CHD had greater likelihood of death in the first year of life than the non-deprived (OR 1.7 [CI 1.4–2.07], I^2^ = 0%) (Figure 4A). Critical CHD patients on government/public insurance also had greater mortality compared to those with private insurance (OR 1.23 [CI 1.08–1.4], I^2^ = 0%) (Figure 4B). Public insurance was associated with increased overall mortality for all forms/ages of CHD compared to private insurance (OR 1.49 [1.39–1.6], I^2^ = 17.4%) (Figure 4C).

#### Maternal Factors (Figure 5)

**FIGURE 5:**
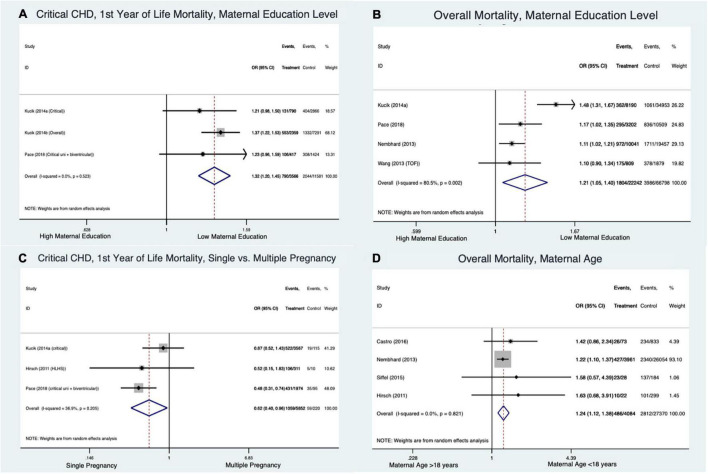
Forest plots demonstrating odds ratio for mortality in congenital heart disease based on maternal factors: maternal education level in critical congenital heart disease in first year of life **(A)**, maternal education level across all ages and forms of congenital heart disease **(B)**, single vs. multiple pregnancy in the first year of life for critical congenital heart disease **(C)**, and maternal age across all congenital heart disease types **(D)**.

Low maternal education did not impact neonatal survival in critical CHD patients (OR 1.09 [CI 0.79–1.51], I^2^ = 42.6%) but it did have an effect on mortality in the first year (OR 1.32 [CI 1.2–1.45], I^2^ = 0%] (Figure 5A). The contribution of maternal education to mortality across all forms/ages of CHD was not clear due to heterogeneity in the data (OR 1.21 [CI 1.05–1.4], I^2^ = 80.5%) (Figure 5B). Singleton pregnancy was associated with a lower mortality in the first year of life from critical CHD compared to multiple pregnancies (OR 0.62 [CI 0.4–0.96], I^2^ = 36.9%) (Figure 5C). Maternal age under 18-20 years was associated with an overall increased odds of death for all CHD (OR 1.24 [CI 1.12–1.38], I^2^ = 0%) (Figure 5D).

#### Hospital Volume and Location (Figure 6)

**FIGURE 6:**
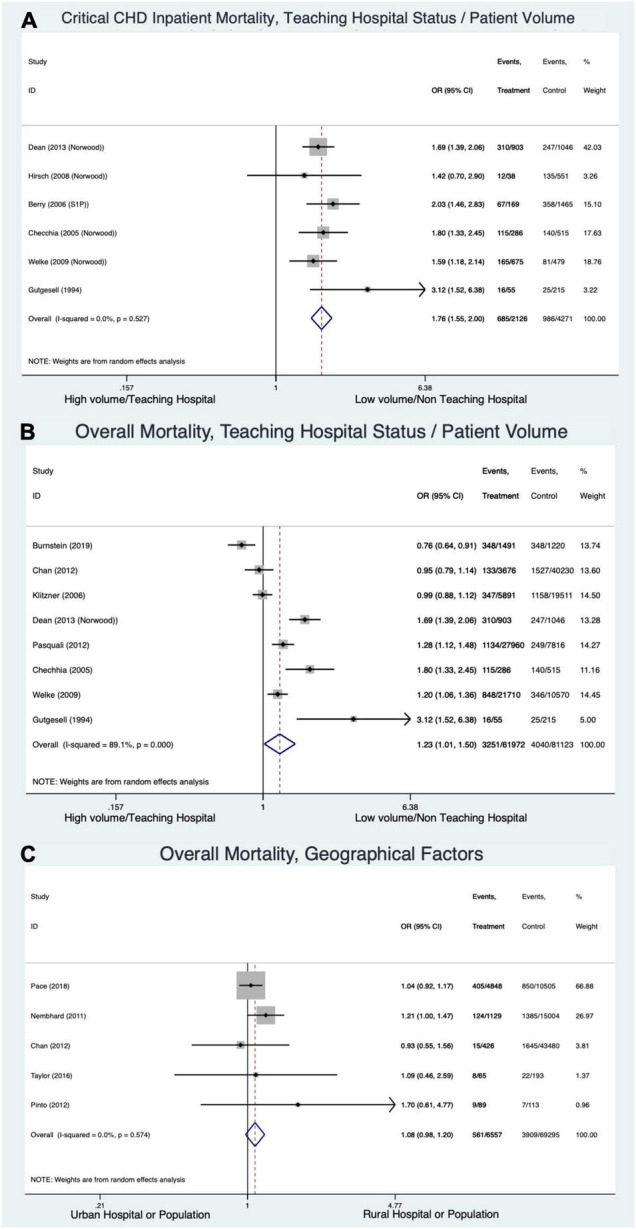
Forest plots demonstrating odds ratio for mortality in congenital heart disease based on hospital volume and location: inpatient mortality in critical congenital heart disease based on volume/teaching status **(A)**, across all forms of congenital heart disease and ages based on volume/teaching status **(B)**, and rural vs. urban hospital **(C)**.

Critical CHD patients at low volume/non-teaching hospitals had significantly higher odds of inpatient mortality than CHD patients at high volume/teaching hospitals (OR 1.76 [CI 1.56–2.0], I^2^ = 0%) (Figure 6A). High heterogeneity did not allow firm conclusions to be drawn on this outcome across all forms/ages of CHD (OR 1.23 [CI 1.01–1.5], I^2^ = 89.1%) (Figure 6B). Rural vs. urban location did not affect overall mortality across all forms of CHD/ages of CHD patients (OR 1.08 [CI 0.98–1.2], I^2^ = 0%) (Figure 6C).

### Other Studies

Insufficient data were available to perform MA for some SDoH. Taylor et al. noted that patients with a single adult caregiver were more likely to have increased interstage mortality following the Norwood procedure (66). This study did not find a relationship between employment status or public assistance status and mortality. Kucik et al. presented data on employment and found no significant relationship with neonatal or infant mortality for seven different types of CHD (55).

A number of individual studies performed multivariable analyses, attempting to understand the modifying as well as confounding effects of different SDoH on CHD mortality. Kucik, et al. presented adjusted hazard ratios (HR) for mortality by race/ethnicity category and concluded that higher infant mortality among the Black patients persisted after adjustment of potential confounding SDoH factors (54). Similarly, Nembhard et al. presented HRs for mortality by racial/ethnic category in Texas, United States (46). They noted that Black children had a significantly increased likelihood of death in the post-neonatal period compared to White children after adjustment for factors such as maternal age, education and residence, infant sex, gestational age and birth weight.

### Risk of Bias

Using the ROBINS-I tool, 14 studies had moderate ROB, 48 serious and three with critical ROB (Supplementary Table 1).

A funnel plot was generated to assess publication bias in studies presenting White versus non-White race/ethnicity as this comprised the largest number of studies in this review (Supplementary Section 3). This funnel plot shows relatively large studies with small standard errors at the apex. There is asymmetry suggesting a publication bias, with smaller studies tending to show a larger effect of non-White race/ethnicity as a risk for CHD mortality.

## Discussion

This SR-MA provides a comprehensive review of the effect of SDoH on risk of mortality stratified across all forms and all ages in patients with CHD. Some trends emerge in this review of 65 studies presenting crude mortality data in 546,981 patients. Racial/ethnic background confers a higher risk of death in Black patients in the first year of life. Familial, social and economic circumstances appear to adversely affect outcomes during specific stages and diagnoses; for example, the MA demonstrated that deprivation status impacts critical CHD mortality outcomes in the first year of life. Another factor that impacts mortality in critical CHD is hospital volume. Rural locations did not confer additional risk of death.

The disparities in outcomes exacerbated by the SDoH at various stages and across all forms of CHD may be addressed in the context of modifiable extrinsic or intrinsic factors as suggested by Ala Szczepura (78). The most commonly reported SDoH in this review was race/ethnicity. As Kaplan and Bennett have noted, it is important to acknowledge that in some cases race/ethnicity may not be the driver behind differences in mortality, but rather that one’s ethnicity is associated with other factors such as place of residence, maternal education and deprivation which may have greater impacts on health (79). A large SR-MA by Paradies et al. has suggested that racial discrimination is a significant determinant of physical and mental health outcomes in the United States (80). While most of the studies in this review are from the United States, this finding is likely to be true in other societies, as is suggested from studies included in our review from countries such as Panama (24) and New Zealand (27).

Racial/ethnic disparities in adult cardiovascular diseases have been described (81). This inequity appears to be present in CHD outcomes as well. Effects of racial/ethnic background on disease progression and mortality need to be investigated and explored. The increased risk for neonatal mortality in Black CHD patients noted in our analysis may perhaps be partially driven by higher rates of prematurity and low birth weight in these communities, both of which are risk factors for poor outcome in critical and single ventricle forms of CHD (82, 83). We also noted that outcomes in the first year of life and thereafter were more likely to be influenced by extrinsic or social and environmental level factors than neonatal outcomes.

Deprivation status of the family likely presents increased financial strain, transportation challenges and food insecurity in vulnerable communities. United States census data highlights the deprivation levels of specific racial/ethnic groups. Black and Hispanic populations have lower median household incomes than White populations (84). Having a child with CHD contributes even further to deprivation and stressors for individuals from these vulnerable communities. A recent investigation by Ludomirsky et al. revealed that families experienced significant financial strain as a result of having a child with CHD (85). Data from the 2009–2010 National Survey of Children with Special Health Care Needs in the United States show that 89.1% of families with a child with CHD experienced at least one financial burden, and 14.9% of caregivers needed mental health services due to the child’s health condition (86). Socioeconomically disadvantaged individuals, especially those living in impoverished neighborhoods, face multiple barriers to availability of healthy, affordable food. An analysis of the US Department of Agriculture data in 2013 showed that 17.5 million United States households (14.3%) and 21% of all children met the definition of a food-insecure household (87). Families of children with CHD are therefore likely to suffer disproportionately as a result of their condition.

Decreased compliance with treatment regimens due to the overwhelming SDoH in vulnerable populations may result in exacerbations or unmet health care needs that worsen outcomes such as during the inter-stage period in high-risk single ventricle patients (88). Lack of health insurance is a major barrier to healthcare. Patients lacking insurance coverage are more likely to avoid doctor visits and fail to adhere to prescribed medications compared to their insured counterparts. Furthermore, there are now clear indications that many adult patients with cardiovascular disease are non-adherent to their much needed medications due to the cost of the medications (89). The scope and determinants of cost related medication non-adherence among the growing CHD population merits further investigation. In parallel, there is a need to develop training programs to enhance healthcare providers’ understanding of the importance of the patient-provider cost conversation while managing these high-risk individuals. Maternal literacy level, age and language fluency likely affect parental understanding of the complexity of their child’s CHD and adherence to medical recommendations and treatment regimens as well. The impact of these maternal-specific SDoH appear to be greatest beyond the neonatal period after patients are discharged from the hospital and when continued follow-up is required. It is known that literacy is a major barrier to optimal health services utilization and a risk factor for poor health outcomes in adult patients. A systematic review of nearly 50 studies found that low health literacy was associated with poor knowledge and understanding of disease condition, chronic disease management and inadequate use of appropriate health services (90). Efforts to improve health literacy should consider educational campaigns and targeted patient/family counseling that may raise awareness for both families and wider population groups.

Intrinsic factors such as cultural differences regarding health beliefs and behaviors in minority ethnic groups may influence outcomes (78). A lack of familiarity with and trust in health services are likely to be an additional barrier to care for immigrants, particularly for those who are undocumented. An implicit bias toward certain racial/ethnic communities among healthcare workers has also been hypothesized to contribute to poor outcomes in CHD patients from ethnic minority backgrounds (15, 78, 91). Future efforts to limit the impact of institutional racism and implicit bias will require further dialogue as well regular education and training of healthcare professionals on the context of racial/ethnic disparities and the best pathways to mitigate these unintended biases in order to provide informed, culturally competent care.

Regionalization of care for CHD has been a topic of much deliberation in the CHD community for many years (74, 92, 93). This SR-MA supports regionalization for critical CHD by demonstrating better outcomes in critical CHD at high volume centers. However, generalized data across all forms of CHD is not as well defined. Perhaps there is a role for lower volume centers continuing to perform non-critical CHD to support the medical and economic needs of local communities and health systems and for continuity of care of patients living in distant locations. Our analysis did not demonstrate poorer outcomes in patients with CHD in rural communities.

### Future Perspective

This SR-MA provides important perspective into the ages and types of CHD that are most likely to be affected by SDoH domains and therefore, a role for focused interventions during those stages of CHD care. A number of interventions and practices could be introduced to achieve equity in CHD outcomes. For example, by increasing research and data collection in routine clinical care, we could further improve our understanding around the impacts of SDoH on numerous other CHD outcomes such as hospitalizations and quality of life. There is an urgent need to implement a more robust and comprehensive patient assessment with tools to identify SDoH and manage those at highest risk of poor outcomes in CHD. Frameworks to create such tools exist (94) and can be expanded to account for a wide array of SDoH.

Tailored services could be developed to improve CHD outcomes among individuals with specific SDoH. The delivery of health services by community health workers (CHW) and medical home model to offer centralized access to CHW resources, care coordination, home visiting and interagency collaboration are attractive options that require further research (95–97). It is likely that many families may have multiple SDoH which put them at higher risk of poor health outcomes, and therefore larger health policy changes would be required to allow for adequate resource allocation and development of the community-level SDoH interventions. Overcoming SDoH barriers in patients with chronic needs will require large health system level innovations to engage communities and multi-pronged interventions at the patient, provider, health system and local and state government levels to address these barriers. There is limited research on interventions for these marginalized communities that can inform widespread policy efforts to improve access to timely, high quality healthcare for CHD patients in order to narrow the racial/ethnic disparities in CHD outcomes highlighted in our review. There is also a great need to provide healthcare workers with the training required to address health disparities and literacy barriers. Finally, systematic research strategies using experimental and quasi-experimental designs will need to be employed to evaluate the impact of SDoH interventions in achieving a change in patient level outcomes.

### Strengths and Limitations

There are several strengths to this analysis. The study followed recommendations on best practice for systematic reviews by registering in PROSPERO, adhering to *a priori* determined project protocol and a methodological robustness in search, extraction and analysis as per the PRISMA Guidelines.

It is unknown if studies had reliable documentation from patients of their race/ethnicity. While this review attempted to categorize SDoH such as race/ethnicity by utilizing clear definitions which best reflected standardized terms recommended in the literature (Table 1 and Supplementary Section 2), it is possible that some studies may have used variations in definitions (98). Many of the SDoH studied lack standardized definitions, posing challenges to analysis. However, this study attempted to create broad definitions to enable analyses and provide an indication of the impact of these SDoH on CHD mortality. It was also not possible to adjust for confounding factors, such as prematurity and low birth weight due to lack of data within studies. Double-counting may have occurred if patients were included in more than one database or registry. However, few analyses contained more than one study from a database/registry and specific efforts were made to avoid inclusion of studies from the same geographical area and timeframe in the same analysis. Only studies that presented crude data were included. It is likely that certain SDoH which are experienced solely as an adult may affect these outcomes differently, but the majority of studies included in this review described pediatric outcomes. The majority of included studies were from the United States. It is likely that SDoH and their associated inequities may be different in other countries. Many studies included in this SR-MA were deemed to have a high RoB, explained by observational study design and that they did not primarily aim for assessment of the SDoH. These studies, therefore, had a higher likelihood of confounding factors that may have influenced mortality outcomes. As such, with appropriate categorization of studies and acknowledgment of RoB, MA was felt to provide the best indication possible of SDoH impacts on CHD mortality and a foundation for future research on this important issue. Careful research examining the direct impact of SDoH on CHD care is necessary to collect and publish the fullest possible data on all potential SDoH variables that could impact mortality. Numerous factors such as food insecurity and housing could not be evaluated due to a lack of literature that directly assessed these factors in patients with CHD.

## Conclusion

This SR-MA provides a unique compilation of all existing SDoH literature that affects CHD mortality outcomes stratified by their complexity and age. We found several disparities in outcomes for racial/ethnic minority patients and vulnerabilities likely aggravated by social risks such as deprivation status, maternal education and age that affect specific time points in the CHD life-course. This analysis will serve as a basis for future research to develop policies and public health programs that can improve CHD outcomes and quality of care, particularly where there is a lack of data and strategies to mitigate existing health inequities in high-risk patients with CHD.

## Disclosures

A part of this SR-MA was presented as the Masters course dissertation thesis by RT at the London School of Economics and Political Science, London, United Kingdom.

## Data Availability Statement

The original contributions presented in the study are included in the article/Supplementary Material, further inquiries can be directed to the corresponding author.

## Author Contributions

RT contributed to the design of the study, literature search, data extraction, data analysis, and writing of the manuscript. RF contributed to the literature search, data extraction, and writing of the manuscript. EM contributed to the theme of the project and edited the manuscript. KN edited the manuscript. AK contributed to the conception and design of the study, as well as writing of the manuscript and providing its strategic direction. All authors contributed to manuscript revision, read, and approved the submitted version.

## Conflict of Interest

The authors declare that the research was conducted in the absence of any commercial or financial relationships that could be construed as a potential conflict of interest.

## Publisher’s Note

All claims expressed in this article are solely those of the authors and do not necessarily represent those of their affiliated organizations, or those of the publisher, the editors and the reviewers. Any product that may be evaluated in this article, or claim that may be made by its manufacturer, is not guaranteed or endorsed by the publisher.
