# Supplementary material for: Social Determinants of Disparities in Mortality Outcomes in Congenital Heart Disease: A Systematic Review and Meta-Analysis
Source: Front Cardiovasc Med. 2022 Mar 15;9:829902. doi: 10.3389/fcvm.2022.829902 (PMC8970097; doi:10.3389/fcvm.2022.829902)
Supplement: Supplementary file 1 [file Data_Sheet_1.DOCX]

SUPPLEMENT SECTION 1. Search Strategy

**OVID Medline Search Terms**

| **#** | **Search Statement** |
| --- | --- |
| 1 | exp "Social Determinants of Health"/ |
| 2 | exp Socioeconomic Factors/ |
| 3 | exp Social Problems/ |
| 4 | exp Gross Domestic Product/ |
| 5 | exp Insurance/ |
| 6 | exp Smoking/ |
| 7 | exp Overnutrition/ |
| 8 | exp Malnutrition/ |
| 9 | exp Diet/ |
| 10 | exp Health Behavior/ |
| 11 | exp Drinking Behavior/ |
| 12 | exp Substance-Related Disorders/ |
| 13 | exp Gender Identity/ |
| 14 | exp Housing/ |
| 15 | exp Residence Characteristics/ |
| 16 | exp Occupations/ |
| 17 | exp Environmental Health/ |
| 18 | exp Homeless Persons/ |
| 19 | exp Sociological Factors/ |
| 20 | exp Religion/ |
| 21 | exp Education/ |
| 22 | exp Literacy/ |
| 23 | Language/ |
| 24 | exp Population Groups/ |
| 25 | exp Human Migration/ |
| 26 | exp Vulnerable Populations/ |
| 27 | exp Family Relations/ |
| 28 | exp Culture/ |
| 29 | exp Social Stigma/ |
| 30 | exp Health Resources/ |
| 31 | exp Environmental Pollution/ |
| 32 | exp Environmental Pollutants/ |
| 33 | social determinant*.tw. |
| 34 | socioeconomic*.tw. |
| 35 | socio-economic*.tw. |
| 36 | smok*.tw. |
| 37 | obes*.tw. |
| 38 | stigma*.tw. |
| 39 | [discrimination.tw](http://discrimination.tw). |
| 40 | [isolation.tw](http://isolation.tw). |
| 41 | [gender.tw](http://gender.tw). |
| 42 | [housing.tw](http://housing.tw). |
| 43 | homeless*.tw. |
| 44 | [rural.tw](http://rural.tw). |
| 45 | urban*.tw. |
| 46 | education*.tw. |
| 47 | [literacy.tw](http://literacy.tw). |
| 48 | illitera*.tw. |
| 49 | (high school adj3 (graduat* or complet* or degree* or drop-out*)).tw. |
| 50 | [sanitation.tw](http://sanitation.tw). |
| 51 | employ*.tw. |
| 52 | unemploy*.tw. |
| 53 | [income.tw](http://income.tw). |
| 54 | [poverty.tw](http://poverty.tw). |
| 55 | [salary.tw](http://salary.tw). |
| 56 | [salaries.tw](http://salaries.tw). |
| 57 | wage*.tw. |
| 58 | occupation*.tw. |
| 59 | ethni*.tw. |
| 60 | [race.tw](http://race.tw). |
| 61 | [insurance.tw](http://insurance.tw). |
| 62 | pollut*.tw. |
| 63 | immigra*.tw. |
| 64 | emigra*.tw. |
| 65 | vulnerable population*.tw. |
| 66 | cultur*.tw. |
| 67 | [nutrition.tw](http://nutrition.tw). |
| 68 | 1 or 2 or 3 or 4 or 5 or 6 or 7 or 8 or 9 or 10 or 11 or 12 or 13 or 14 or 15 or 16 or 17 or 18 or 19 or 20 or 21 or 22 or 23 or 24 or 25 or 26 or 27 or 28 or 29 or 30 or 31 or 32 or 33 or 34 or 35 or 36 or 37 or 38 or 39 or 40 or 41 or 42 or 43 or 44 or 45 or 46 or 47 or 48 or 49 or 50 or 51 or 52 or 53 or 54 or 55 or 56 or 57 or 58 or 59 or 60 or 61 or 62 or 63 or 64 or 65 or 66 or 67 |
| 69 | exp Mortality/ |
| 70 | exp Prognosis/ |
| 71 | exp Death/ |
| 72 | exp Survival/ |
| 73 | [mortality.tw](http://mortality.tw). |
| 74 | [prognosis.tw](http://prognosis.tw). |
| 75 | [death.tw](http://death.tw). |
| 76 | [survival.tw](http://survival.tw). |
| 77 | 69 or 70 or 71 or 72 or 73 or 74 or 75 or 76 |
| 78 | exp Heart Defects, Congenital/ |
| 79 | Heart Diseases/cn [Congenital] |
| 80 | congenital [heart.tw](http://heart.tw). |
| 81 | congenital [cardiac.tw](http://cardiac.tw). |
| 82 | [guch.tw](http://guch.tw). |
| 83 | septal [defect.tw](http://defect.tw). |
| 84 | patent ductus [arteriosus.tw](http://arteriosus.tw). |
| 85 | (transposition adj4 arteries).tw. |
| 86 | (transposition adj4 vessels).tw. |
| 87 | (fallot* adj3 tetralogy).tw. |
| 88 | (interrupt* adj3 aort*).tw. |
| 89 | (hypoplastic adj3 heart).tw. |
| 90 | (coarctation adj3 aort*).tw. |
| 91 | (atresia adj3 pulmonary).tw. |
| 92 | (atresia adj3 aort*).tw. |
| 93 | double outlet right [ventricle.tw](http://ventricle.tw). |
| 94 | [dorv.tw](http://dorv.tw). |
| 95 | atrioventricular septal [defect.tw](http://defect.tw). |
| 96 | [avsd.tw](http://avsd.tw). |
| 97 | [vsd.tw](http://vsd.tw). |
| 98 | [asd.tw](http://asd.tw). |
| 99 | cyanotic [heart.tw](http://heart.tw). |
| 100 | acyanotic [heart.tw](http://heart.tw). |
| 101 | univentricular [heart.tw](http://heart.tw). |
| 102 | truncus [arteriosus.tw](http://arteriosus.tw). |
| 103 | ebstein* [anomaly.tw](http://anomaly.tw). |
| 104 | 78 or 79 or 80 or 81 or 82 or 83 or 84 or 85 or 86 or 87 or 88 or 89 or 90 or 91 or 92 or 93 or 94 or 95 or 96 or 97 or 98 or 99 or 100 or 101 or 102 or 103 |
| 105 | 68 and 77 and 104 |

**OVID Embase Search Terms**

| **#** | **Search Statement** |
| --- | --- |
| 1 | exp "social determinants of health"/ |
| 2 | exp "social aspects and related phenomena"/ |
| 3 | exp gross national product/ |
| 4 | exp insurance/ |
| 5 | exp "tobacco use"/ |
| 6 | exp overnutrition/ |
| 7 | exp malnutrition/ |
| 8 | exp health behavior/ |
| 9 | exp drug dependence/ |
| 10 | exp "gender and sex"/ |
| 11 | exp housing/ |
| 12 | exp demography/ |
| 13 | exp occupation/ |
| 14 | exp environmental health/ |
| 15 | exp sanitation/ |
| 16 | exp homeless person/ |
| 17 | exp homelessness/ |
| 18 | exp religion/ |
| 19 | exp education/ |
| 20 | exp educational status/ |
| 21 | exp population group/ |
| 22 | language/ |
| 23 | exp migration/ |
| 24 | exp vulnerable population/ |
| 25 | exp family relation/ |
| 26 | exp cultural anthropology/ |
| 27 | exp stigma/ |
| 28 | exp social stigma/ |
| 29 | exp health care planning/ |
| 30 | exp pollution/ |
| 31 | exp pollutant/ |
| 32 | social determinant*.tw. |
| 33 | socioeconomic*.tw. |
| 34 | socio-economic*.tw. |
| 35 | smok*.tw. |
| 36 | obes*.tw. |
| 37 | stigma*.tw. |
| 38 | [discrimination.tw](http://discrimination.tw). |
| 39 | [isolation.tw](http://isolation.tw). |
| 40 | [gender.tw](http://gender.tw). |
| 41 | [housing.tw](http://housing.tw). |
| 42 | homeless*.tw. |
| 43 | [rural.tw](http://rural.tw). |
| 44 | urban*.tw. |
| 45 | education*.tw. |
| 46 | [literacy.tw](http://literacy.tw). |
| 47 | illitera*.tw. |
| 48 | (high school adj3 (graduat* or complet* or degree* or drop-out*)).tw. |
| 49 | [sanitation.tw](http://sanitation.tw). |
| 50 | employ*.tw. |
| 51 | unemploy*.tw. |
| 52 | [income.tw](http://income.tw). |
| 53 | [poverty.tw](http://poverty.tw). |
| 54 | [salary.tw](http://salary.tw). |
| 55 | [salaries.tw](http://salaries.tw). |
| 56 | wage*.tw. |
| 57 | occupation*.tw. |
| 58 | ethni*.tw. |
| 59 | [race.tw](http://race.tw). |
| 60 | [insurance.tw](http://insurance.tw). |
| 61 | pollut*.tw. |
| 62 | immigra*.tw. |
| 63 | emigra*.tw. |
| 64 | vulnerable population*.tw. |
| 65 | cultur*.tw. |
| 66 | [nutrition.tw](http://nutrition.tw). |
| 67 | 1 or 2 or 3 or 4 or 5 or 6 or 7 or 8 or 9 or 10 or 11 or 12 or 13 or 14 or 15 or 16 or 17 or 18 or 19 or 20 or 21 or 22 or 23 or 24 or 25 or 26 or 27 or 28 or 29 or 30 or 31 or 32 or 33 or 34 or 35 or 36 or 37 or 38 or 39 or 40 or 41 or 42 or 43 or 44 or 45 or 46 or 47 or 48 or 49 or 50 or 51 or 52 or 53 or 54 or 55 or 56 or 57 or 58 or 59 or 60 or 61 or 62 or 63 or 64 or 65 or 66 |
| 68 | exp mortality/ |
| 69 | exp prognosis/ |
| 70 | exp death/ |
| 71 | exp survival/ |
| 72 | [mortality.tw](http://mortality.tw). |
| 73 | [prognosis.tw](http://prognosis.tw). |
| 74 | [death.tw](http://death.tw). |
| 75 | [survival.tw](http://survival.tw). |
| 76 | 68 or 69 or 70 or 71 or 72 or 73 or 74 or 75 |
| 77 | exp congenital heart disease/ |
| 78 | heart disease/cn [Congenital Disorder] |
| 79 | congenital [heart.tw](http://heart.tw). |
| 80 | congenital [cardiac.tw](http://cardiac.tw). |
| 81 | [guch.tw](http://guch.tw). |
| 82 | septal [defect.tw](http://defect.tw). |
| 83 | patent ductus [arteriosus.tw](http://arteriosus.tw). |
| 84 | (transposition adj4 arteries).tw. |
| 85 | (transposition adj4 vessels).tw. |
| 86 | (fallot* adj3 tetralogy).tw. |
| 87 | (interrupt* adj3 aort*).tw. |
| 88 | (hypoplastic adj3 heart).tw. |
| 89 | (coarctation adj3 aort*).tw. |
| 90 | (atresia adj3 pulmonary).tw. |
| 91 | (atresia adj3 aort*).tw. |
| 92 | double outlet right [ventricle.tw](http://ventricle.tw). |
| 93 | [dorv.tw](http://dorv.tw). |
| 94 | atrioventricular septal [defect.tw](http://defect.tw). |
| 95 | [avsd.tw](http://avsd.tw). |
| 96 | [vsd.tw](http://vsd.tw). |
| 97 | [asd.tw](http://asd.tw). |
| 98 | cyanotic [heart.tw](http://heart.tw). |
| 99 | acyanotic [heart.tw](http://heart.tw). |
| 100 | univentricular [heart.tw](http://heart.tw). |
| 101 | truncus [arteriosus.tw](http://arteriosus.tw). |
| 102 | ebstein* [anomaly.tw](http://anomaly.tw). |
| 103 | 77 or 78 or 79 or 80 or 81 or 82 or 83 or 84 or 85 or 86 or 87 or 88 or 89 or 90 or 91 or 92 or 93 or 94 or 95 or 96 or 97 or 98 or 99 or 100 or 101 or 102 |
| 104 | 67 and 76 and 103 |

**Web of Science Search Terms**

|  | **Search Statement** |
| --- | --- |
| # 4 | #3 AND #2 AND #1 |
| # 3 | TS=("congenital heart disease") OR TS=("congenital heart defect") OR TS=("congenital cardiac disease") OR TS=("congenital cardiac defect") OR TS=(guch) OR TS=("septal defect") OR TS=(asd) OR TS=(vsd) OR TS=(avsd) OR TS=("patent ductus arteriosus") OR TS=("transposition of the great arteries") OR TS=("transposition of the great vessels") OR TS=("tetralogy of fallot") OR TS=("fallot's tetralogy") OR TS=("interrupted aortic arch") OR TS=("hypoplastic left heart") OR TS=("coarctation of the aorta") OR TS=("aortic coarctation") OR TS=("pulmonary atresia") OR TS=("aortic atresia") OR TS=("double outlet right ventricle") OR TS=("dorv") OR TS=("cyanotic heart") OR TS=("acyanotic heart") OR TS=("univentricular heart") OR TS=("univentricular circulation") OR TS=("truncus arteriosus") OR TS=("ebstein anomaly") OR TS=("ebstein's anomaly") |
| # 2 | TS=(mortality) OR TS=(prognosis) OR TS=(death) OR TS=(survival) |
| # 1 | TS=("social determinants of health") OR TS=(socioeconomic) OR TS=(socio-economic) OR TS=("gross domestic product") OR TS=("health insurance") OR TS=(smok*) OR TS=(obes*) OR TS=(malnutrition) OR TS=(diet) OR TS=("health behaviour") OR TS=("health behavior") OR TS=(Alcohol*) OR TS=(addict*) OR TS=(gender) OR TS=(housing) OR TS=(occupation) OR TS=(employ*) OR TS=(unemploy*) OR TS=(environment*) OR TS=(homeless*) OR TS=(sociological) OR TS=(religion) OR TS=(education) OR TS=(literacy) OR TS=(illitera*) OR TS=(ethnic*) OR TS=(migration) OR TS=(immigra*) OR TS=(emigra*) OR TS=(race) OR TS=(family) OR TS=(culture) OR TS=(stigma) OR TS=(discrimination) OR TS=(isolation) OR TS=("health resources") OR TS=(pollut*) OR TS=(rural) OR TS=(urban) OR TS=(sanitation) OR TS=(income) OR TS=(salar*) OR TS=(wage*) OR TS=(poverty) |

**CINAHL Search Terms**

| **Concept 1** |
| --- |
| MH “Social Determinants of Health” |
| OR |
| MH “Socioeconomic factors+” |
| OR |
| “MH Social Problems+” |
| OR |
| MH “Insurance+” |
| OR |
| MH “Smoking+” |
| OR |
| MH “Obesity+” |
| OR |
| MH “Malnutrition” |
| OR |
| MH “Diet+” |
| OR |
| MH “Health Behavior+” |
| OR |
| MH “Drinking Behavior+” |
| OR |
| MH “Substance Use Disorders+” |
| OR |
| MH “Gender Identity+” |
| OR |
| MH “Residence Characteristics+” |
| OR |
| MH “Occupations and Professions+” |
| OR |
| MH “Environment+” |
| OR |
| MH “Sanitation+” |
| OR |
| MH “Homeless Persons” |
| OR |
| MH “Sociology+” |
| OR |
| MH “Religion and Religions+” |
| OR |
| MH “Education+” |
| OR |
| MH “Ethnic Groups+” |
| OR |
| MH “Residential Mobility+” |
| OR |
| MH “Refugees” |
| OR |
| MH “Family Relations+” |
| OR |
| MH “Culture+” |
| OR |
| MH “Stigma” |
| OR |
| MH “Social Identity+” |
| OR |
| MH “Resource Allocation+” |
| OR |
| MH “Environmental Pollution+” |
| OR |
| MH “Environmental Pollutants+” |
| OR |
| TX social determinant* |
| OR |
| TX socioeconomic* |
| OR |
| TX socio-economic* |
| OR |
| TX smok* |
| OR |
| TX obes* |
| OR |
| TX stigma* |
| OR |
| TX discrimination |
| OR |
| TX isolation |
| OR |
| TX gender |
| OR |
| TX housing |
| OR |
| TX homeless* |
| OR |
| TX rural |
| OR |
| TX urban* |
| OR |
| TX education* |
| OR |
| TX literacy |
| OR |
| TX illitera* |
| OR |
| TX (high school N3 (graduat* OR complet* OR degree* OR drop-out*)) |
| OR |
| TX sanitation |
| OR |
| TX employ* |
| OR |
| TX unemploy* |
| OR |
| TX income |
| OR |
| TX poverty |
| OR |
| TX salary |
| OR |
| TX salaries |
| OR |
| TX wage* |
| OR |
| TX occupation* |
| OR |
| TX ethni* |
| OR |
| TX race |
| OR |
| TX insurance |
| OR |
| TX pollut* |
| OR |
| TX immigra* |
| OR |
| TX emigra* |
| OR |
| TX vulnerable population* |
| OR |
| TX cultur* |
|  |
| **Concept 2** |
| (Mortality |
| OR |
| Prognosis |
| OR |
| Death |
| OR |
| Survival |
| OR |
| TX prognosis |
| OR |
| TX mortality |
| OR |
| TX death |
| OR |
| TX survival |
|  |
| **Concept 3** |
| (Heart Defects, Congenital |
| OR |
| TX congenital heart |
| OR |
| TX congenital cardiac |
| OR |
| TX guch |
| OR |
| TX coarctation N3 aort* |
| OR |
| TX septal defect |
| OR |
| TX patent ductus arteriosus |
| OR |
| TX transposition N4 arteries |
| OR |
| TX transposition N4 vessels |
| OR |
| TX tetralogy N3 fallot* |
| OR |
| TX interrupt* N3 aort* |
| OR |
| TX hypoplastic N3 heart |
| OR |
| TX pulmonary N3 atresia |
| OR |
| TX aort* N3 atresia |
| OR |
| TX double outlet right ventricle |
| OR |
| TX atrioventricular septal defect |
| OR |
| TX avsd |
| OR |
| TX cyanotic heart |
| OR |
| TX acyanotic heart |
| OR |
| TX univentricular heart |
| OR |
| TX truncus arteriosus |
| OR |
| TX ebstein* anomaly |
| OR |
| TX vsd |
| OR |
| TX asd |
| OR |
| TX dorv |
|  |
| **Concept 1 AND Concept 2 AND Concept 3** |

SUPPLEMENT SECTION 2.

**Social Determinants of Health – Definitions of Analysis Categories**

This document provides a list of definitions for the analysis categories that were used in this Systematic Review & Meta-Analysis.

**Race/Ethnicity**

For the purposes of analysis in our study, we used standardized definition of ‘race/ethnicity’ as per US census data (<https://www.census.gov/topics/population/race/about.html>) as follows: White, Black, Hispanic, and Other (which included all Asian ethnicities). The analysis is limited by the identification of racial/ethnic backgrounds as described in the original included studies, that may be varied. For example, some studies such as Nembhard 2013 classified by maternal race/ethnicity, which may not provide a complete representation of the patient’s true classification. Studies did not specify Black participants as Hispanic or non-Hispanic, but for the purposes of data extraction and analysis, all Black patients were categorized as such without knowledge of their ethnicity. It is also not known if patients self- identified or were identified by other means into each racial/ethnic category in the included studies. The authors of this study acknowledge that these categories are quite broad, and because of this, there may be variation of health outcomes of individuals within these categories. Furthermore, the authors recognize that racial disparities are likely different in different countries.

| **US Census Bureau Race Classification** | **US Census Bureau Definition** | **Classification used in our study** |
| --- | --- | --- |
| White | A person having origins in any of the original peoples of Europe, the Middle East, or North Africa. | White |
| Black or African American | A person having origins in any of the Black racial groups of Africa. | Black |
| American Indian or Alaska Native | A person having origins in any of the original peoples of North and South America (including Central America) and who maintains tribal affiliation or community attachment. | Other  *N.B. Aboriginals and other native peoples across different countries fell into this category |
| Asian | A person having origins in any of the original peoples of the Far East, Southeast Asia, or the Indian subcontinent including, for example, Cambodia, China, India, Japan, Korea, Malaysia, Pakistan, the Philippine Islands, Thailand, and Vietnam. | Other |
| Native Hawaiian or Other Pacific Islander | A person having origins in any of the original peoples of Hawaii, Guam, Samoa, or other Pacific Islands. | Other |
|  | According to the CDC, People who identify their origin as Hispanic, Latino, or Spanish may be of any race. For the purposes of this study, there was a separate category for Hispanic. | Hispanic |

**Deprivation**

Definitions of deprivation varied between the included studies; they used either household income thresholds, census data for percentage of the population in poverty, or pre-defined deprivation indices. Studies presented deprivation data as ‘most versus least deprived’ (e.g. population poverty <5% versus >20% OR high versus low median household income) or ‘most deprived versus the rest’ (e.g. population poverty <20% versus >20%). We analyzed and categorized deprivation status for the MA as follows:

- Studies were included that classified the study population either by income or by poverty/deprivation level;
- Definitions of deprivation or household income thresholds as presented in any given study were used as such;
- Comparisons were made between the most and least deprived categories where possible. If a study only provided most deprived versus the rest of the population, these data were also categorized into most deprived and least deprived.

**Insurance Status**

For the purposes of analysis, patients were divided into public and private insurance categories as described in the table below. Public insurance included those patients listed in any given study as having ‘government insurance’ as well as ‘state-funded healthcare insurance’, such as Medicaid in the USA. Medicare was variably listed as either ‘public’ or ‘other’ insurance in different studies. Private insurance included those listed as having ‘managed care’.

| **Insurance Classification in Study Reviewed** | **Classification used in our study** |
| --- | --- |
| Private / Commercial | Private |
| Managed Care |  |
| Medicare | Public |
| Medicaid |  |
| Government |  |
| Public |  |
| Uninsured | No insurance |
| Other | Other |

**Maternal education**

Maternal education was classified as being either ‘low’ or ‘high’ in our analysis. Center for Disease Control (CDC) maternal education definitions (<https://wonder.cdc.gov/wonder/help/natality.html#Education>) were used to create a categorisation as follows:

- Low maternal education = Completed <12 years of education with no diploma;
- High maternal education = Completed 12 years or more years of education, High school graduate or GED completed, some college credit or higher degree.

**Single / Multiple pregnancy**

Multiple pregnancy category included both twin and/ multiple pregnancies

**Maternal age**

Maternal age ranges did not always coincide exactly between included studies. For the purposes of this analysis, patients were categorized into maternal age <18/20 years and >18/20 years and patients were classified into whichever of the age ranges was closest.

**Hospital Volume and Skill Level**

A broad MA using combined volume or teaching hospital status was performed.

Hospital case volume definitions were not homogenous between studies. Klitzner 2006 classed high volume as >100 cases/year, Pasquali 2012 classed high volume as >350 cases/year and Dean 2013 presented the “Top 5” versus “non-Top 5” institutions in respect to institutional volume over a 10 year period. We chose to use the same categories as low vs high volume centers as originally described in these studies for the MA and did not categorize it further.

Three studies presented the hospitals as either teaching or non-teaching hospital. All these studies used data from the HCUP KID inpatient database. One of these studies – Berry 2006 – states: “In the KID, teaching designation is obtained from the American Hospital Association (AHA) Annual Survey of Hospitals. Teaching hospitals have an American Medical Association-approved residency program or a membership in the Council of Teaching Hospitals. The KID 2000 also recognizes a full-time intern and resident to bed ratio of 0.25 or higher as a teaching hospital criteria. The KID does not contain the residency program type(s) associated with teaching hospitals.” It was not possible to present MA on this category alone due to potential overlapping of the studies.

**Geographical location of hospital or patient population**

For the purposes of analysis, patients were divided into urban versus rural categories. Studies used various definitions of geographical location, with some describing the hospital location and others describing the location of the population who underwent cardiac surgery. Others used driving time to the cardiac centre or nearest paediatric intensive care unit. Our MA accepted individual study definitions of rural / urban patient population or hospital location and classified driving time or distance to the nearest specialist facility as urban or rural (shortest driving time/distance = urban; longest driving time/distance = rural).

# SUPPLEMENT SECTION 3. Funnel Plot for Race/Ethnicity Studies
