# Supplementary material for: Social Determinants of Disparities in Mortality Outcomes in Congenital Heart Disease: A Systematic Review and Meta-Analysis
Source: Front Cardiovasc Med. 2022 Mar 15;9:829902. doi: 10.3389/fcvm.2022.829902 (PMC8970097; doi:10.3389/fcvm.2022.829902)
Supplement: Supplementary file 2 [file Table_1.doc]

Supplementary Table. Characteristics of included studies

| **Citation No:** | **First Author** | **Year** | **Journal** | **Country** | **Database (where applicable)** | **SDoH Reported** | **Duration of Study** | **Primary type of CHD or procedure studied** | **Total number patients with CHD*** | **CHD Deaths*** | **Age at enrolment / diagnosis / surgery** | **Timing of mortality studied** | **Risk of Bias** |
| --- | --- | --- | --- | --- | --- | --- | --- | --- | --- | --- | --- | --- | --- |
| **1** | Alsaied | 2017 | World Journal for Pediatric and Congenital Heart Surgery | USA | Organ Procurement and Transplant Network | Ethnicity | 1990-2005 | Heart transplantation | 1265 | 426 | <1year - 17 years | One-year conditional ten-year survival | Serious |
| **2** | Chan | 2018 | The Journal of Thoracic and Cardiovascular Surgery | USA | Pediatric Health Information System (PHIS) | Ethnicity | 2004-2015 | ECMO use following cardiac surgery | 130860** | 4254 | <29 days - >12 years | Inpatient mortality following surgery | Serious |
| **3** | Burnstein† | 2019 | American Heart Journal | USA | PHIS | Case volume; Ethnicity | 2004-2015 | Advanced heart failure in CHD | 2712** | 688 | <1year - 21 years | Inpatient mortality | Serious |
| **4** | Oster† | 2011a | The Journal of Pediatrics | USA | PHIS | Ethnicity; Insurance status | 2004-2008 | Various | 44017** | 1694 | <18 years | Inpatient mortality following surgery | Serious |
| **5** | Oster† | 2011b | The Journal of Thoracic and Cardiovascular Surgery | USA | PHIS | Ethnicity; Insurance status | 2004-2008 | Various | 24112** | 774 | <18 years | Inpatient mortality following surgery | Serious |
| **6** | Anderson | 2018 | Pediatrics | USA | PHIS | Deprivation / Income | 2005 – 2015 | Various | 101013** | 2933 | <19 years | Inpatient mortality following surgery | Serious |
| **7** | Puri | 2018 | Surgery | USA | PHIS | Ethnicity | 2004-2015 | Ductal dependent CHD with oesophageal atresia / tracheo-esophageal fistula | 496 (124 with oesophageal atresia / tracheo-esophageal fistula) | 55 (25 of those with oesophageal abnormality) | Neonates | Inpatient mortality | Serious |
| **8** | Prodhan | 2017 | Annals of Thoracic Surgery | USA | PHIS | Ethnicity | 2004-2013 | Patients with HLHS and tracheostomy | 126 | 33 | Neonates | Inpatient mortality following palliative operation or heart transplantation | Serious |
| **9** | Kim | 2011 | Circulation. Cardiovascular Quality and Outcomes | USA | PHIS | Ethnicity; Insurance status | 2000-2008 | Various | 3061** | 67 | 18-49 years | Inpatient mortality | Serious |
| **10** | Checchia† | 2005 | The Journal of Thoracic and Cardiovascular Surgery | USA | PHIS | Case volume | 1998-2001 | HLHS | 801 | 255 | Neonates | 28 day survival after Norwood Procedure | Serious |
| **11** | Castro† | 2016 | PLoS ONE | Panama | N/A | Ethnicity; Maternal age; Maternal education | 2010-2014 | Various | 954 | 284 | < 1 year | First year of life | Serious |
| **12** | Chan | 2017 | Critical Care Medicine | International | Extracorporeal Life Support Organization (ELSO) International Registry | Ethnicity | 1998-2012 | ECMO in CHD patients | 5338 | 2921 | <29 days – 18 years | Inpatient mortality | Serious |
| **13** | Sherwin | 2012 | The Journal of Thoracic and Cardiovascular Surgery | International | ELSO International Registry | Ethnicity | 2000-2009 | ECMO after stage 1 palliation for HLHS | 738 | 512 | Neonates | Inpatient mortality following surgery | Serious |
| **14** | Cloete | 2019 | Archives of Disease in Childhood | New Zealand | National cardiac surgery and cardiology databases | Ethnicity | 2006-2014 | Left heart obstruction (HLHS, AAO, AVSA) | 171 (terminations and stillbirths excluded) | 48 | Neonates | First year of life | Serious |
| **15** | Crowe | 2016 | Journal of the American Heart Association | England & Wales | National Congenital Heart Disease Audit (NCHDA) and Paediatric Intensive Care Audit Network (PICANet) | Deprivation / Income; Ethnicity | 2005-2010 | Various | 7643 | 246 | 0 days - >3 months | Within one year of discharge following intervention in infancy | Moderate |
| **16** | Knowles | 2019 | Archives of Disease in Childhood | England & Wales | NCHDA and PICANet | Ethnicity | 2006-2009 | Various | 5350 | 449 | <1 year | Primary: First year of life. Secondary: Death during index hospital admission; death outside hospital or after urgent ICU admission; death during elective readmission | Serious |
| **17** | Dean | 2013 | Pediatric Cardiology | USA | University Health System Consortium | Case volume; Ethnicity | 1998-2007 | HLHS (separate data given on 3 palliative stages) | 1949 | 670 | <1 year | Inpatient mortality following surgery | Serious |
| **18** | DiBardino | 2012 | The Annals of Thoracic Surgery | USA | STS Congenital Heart Surgery Database (STS-CHSD) | Ethnicity | 2007-2009 | Various | 20399 | 866 | <18 years | Inpatient mortality following surgery | Serious |
| **19** | Gonzalez† | 2003 | Pediatric Cardiology | USA | Four US State (CA, MA, NY, PA) Databases containing hospital discharge abstract data | Ethnicity | 1996 | Various | 4822** | 197 | <18 years | Inpatient mortality following surgery | Serious |
| **20** | Demone† | 2003 | Pediatric Cardiology | USA | Administrative data from five US States (CA, MA, IL, PA, WA) | Insurance status | 1996 | Various | 4729** | 227 | <18 years | Inpatient mortality following surgery | Serious |
| **21** | Erickson† | 2000 | Pediatrics | USA | California state-mandated hospital discharge data | Insurance status | 1992-1994 | Various | 5071 | 325 | <15 years | Inpatient mortality | Serious |
| **22** | Ghanayem | 2012 | Congenital Heart Disease | USA & Canada | N/A | Deprivation / Income; Ethnicity | 2005-2008 | Norwood procedure | 426 | 50 | Neonates | Interstage mortality after the Norwood procedure | Serious |
| **23** | Chan | 2012 | Pediatric Cardiology | USA | Healthcare Cost and Utilization Project (HCUP) Kids' Inpatient Database (KID) | Teaching / non-teaching hospital; Ethnicity; Insurance status; Geographical location. | 1997, 2000, 2003, 2006 | Various | 44910** | 1709 | <18 years | Inpatient mortality following surgery | Serious |
| **24** | Benavidez | 2006 | Pediatric Cardiology | USA | HCUP KID | Race / Ethnicity | 2000 | Various | 8483** | 348 | <18 years | Inpatient mortality following surgery | Moderate |
| **25** | Seifert | 2007 | The Journal of Thoracic and Cardiovascular Surgery | USA | HCUP KID | Case volume; Deprivation / Income; Ethnicity; Insurance status | 2000 | Various | 10282** | 474 | <20 years | Inpatient mortality following surgery | Moderate |
| **26** | Hirsch | 2008 | Pediatric Cardiology | USA | HCUP KID | Teaching / non-teaching hospital; Ethnicity; Geographical location. | 2003 | HLHS or TGA | 1171 | 186 | <1 year | Inpatient mortality | Serious |
| **27** | Berry† | 2006 | Pediatrics | USA | HCUP KID | Teaching / non-teaching hospital | 1997, 2000 | HLHS | 1634 | 422 | Neonates | Inpatient mortality | Serious |
| **28** | Peterson† | 2017 | Congenital Heart Disease | USA | HCUP KID | Ethnicity | 2012 | Various | 13130** | 391 | <18 years | Inpatient mortality following surgery | Serious |
| **29** | Hirsch | 2011 | The Journal of Pediatrics | USA | Michigan Birth Defects Registry | Deprivation / Income; Ethnicity; Insurance status; Maternal age; Single / Multiple Pregnancy | 1992-2005 | HLHS | 321 | 111 | Neonates | First year of life | Moderate |
| **30** | Mat Bah | 2018 | Pediatric Cardiology | Malaysia | Pediatric Cardiology Clinical Information System | Ethnicity | 2006-2015 | Various “Critical” CHD | 491 | 171 | <1 year | Median age of death 2.7 months (IQR 1-7.3 months) | Serious |
| **31** | Zahari | 2019 | European Journal of Pediatrics | Malaysia | N/A | Ethnicity | 2006-2015 | CHD in patients with Trisomy 21 (mostly AVSD, VSD or PDA) | 414 | 93 | Median age at time of diagnosis 7 days | Median follow-up 2.7 years; median age of death 7.6 months | Serious |
| **32** | Nembhard | 2016 | Birth Defects Research (Part A) | Australia | Western Australian Register of Developmental Anomalies | Ethnicity | 1980-2010 | Various | 4339 | 698 | Neonates | Long term (25 year) mortality | Serious |
| **33** | Nembhard | 2013 | Birth Defects Research (Part A) | USA | Texas Birth Defects Registry | Ethnicity; Maternal age; Maternal education | 1999-2008 | Various | 30015 | 2767 | Neonates | Up to 10 years of age | Serious |
| **34** | Lara | 2016 | Birth Defects Research (Part A) | USA | Texas Birth Defects Registry | Ethnicity; Geographical location; Maternal age; Maternal education; Single / Multiple Pregnancy | 1999-2007 | TGA | 468 | 20 | Neonates | Neonatal mortality | Serious |
| **35** | Morris | 2014 | Circulation | USA | Texas Birth Defects Registry | Deprivation / Income; Ethnicity; Geographical location; Maternal education | 1999-2007 | HLHS | 463 | 123 | Neonates | Neonatal mortality | Moderate |
| **36** | Nembhard | 2011 | Pediatrics | USA | Texas Birth Defects Registry | Ethnicity; Geographical location; Maternal age; Maternal education | 1996-2003 | Various | 19530 | 1826 | Neonates | During ~10 year follow-up period | Serious |
| **37** | Fixler† | 2012 | Pediatrics | USA | Texas Birth Defects Registry | Ethnicity | 1996-2003 | Various “Severe” CHD | 1213 | 486 | Neonates | First year of life | Serious |
| **38** | Ingaramo | 2012 | Pediatric Critical Care Medicine | USA | Virtual PICU Performance System | Ethnicity | 2006-2008 | HLHS, TA, Common Ventricle | 423 | 6 | Bi-directional Glenn: <1 year | Perioperative mortality | Serious |
| **39** | Justo† | 2017 | Cardiology in the Young | Australia | N/A | Ethnicity | 2006-2014 | Various | 1528 | 24 | Mean 3.4 years | Perioperative mortality | Moderate |
| **40** | Klass | 1990 | South African Medical Journal | South Africa | N/A | Ethnicity | 1975-1985 | VSD | 309 | 17 | 0 months - >6 years | Within 1 month of surgery | Critical |
| **41** | Kucik | 2014a | American Journal of Public Health | USA | Florida Birth Defects Registry | Ethnicity; Insurance status; Maternal age; Maternal education; Single / Multiple Pregnancy | 1998-2007 | Various – divided into Critical and Noncritical | 43411 | 1443 | <1 year | First year of life | Moderate |
| **42** | Kucik | 2014b | American Journal of Public Health | USA | Four US State Databases (Arizona, New Jersey, New York, Texas) | Deprivation; Ethnicity; Maternal age; Maternal education; Parental occupation | 1999-2007 | Various | 9853 | 1942 | <1 year | Deaths in neonatal (<28d), post neonatal (28-364d) and overall infant (0-364d) periods. | Moderate |
| **43** | Kuehl† | 2000 | Journal of Health Care for the Poor and Underserved | USA | Baltimore Washington Infant Study | Insurance status | 1981-1989 | Coarctation of the Aorta | 105 | 9 | <1 year | Majority of deaths in first year of life | Serious |
| **44** | Klitzner | 2006 | Congenital Heart Disease | USA | California Office of Statewide Health Planning and Development (OSHPD) | Case volume; Deprivation / Income; Ethnicity; Insurance status | 1989-1999 | Various | 25402 | 1505 | <18 years | Inpatient mortality | Moderate |
| **45** | Chang | 2006 | American Heart Journal | USA | OSHPD | Case volume; Deprivation / Income; Ethnicity; Insurance status | 1989-1999 | Various | 23897** | 148 | <18 years | Within 30 days and 1 year after hospital discharge for cardiac surgery | Moderate |
| **46** | Chang | 2002 | Circulation | USA | OSHPD | Case volume; Deprivation / Income; Ethnicity; Insurance status | 1995-1997 | Various | 6593 | 345 | <21 years | Inpatient mortality following surgery | Moderate |
| **47** | Peyvandi | 2018 | Journal of the American Heart Association | USA | OSHPD | Ethnicity | 2007-2012 | HLHS or TGA | 1796 | 251 | Neonates | First year of life; Mortality before discharge, Mortality after discharge. | Serious |
| **48** | Lasa† | 2013 | Pediatric Cardiology | USA | N/A | Ethnicity | 2005-2006 | Various | 217 | 16 | Neonates | Mortality following discharge after surgery. Mean follow-up time 23.9 months. | Serious |
| **49** | Pinto† | 2012 | Pediatric Cardiology | USA | N/A | Geographical location | 2005-2006 | Various | 217 | 16 | Neonates | Mortality following discharge after surgery. Mean follow-up time 23.9 months. | Serious |
| **50** | Milazzo | 2002 | Journal of the American Medical Association | USA | N/A | Ethnicity | 1997-2000 | Bi-directional Glenn and Fontan procedures (data presented separately) | 40 | 9 | Bi-directional Glenn: Majority <1 year. Fontan: 1 year - >12 years | Inpatient mortality following surgery | Serious |
| **51** | Pace† | 2018 | Pediatrics | USA | North Carolina Birth Defects Monitoring Program (NCBDMP) | Deprivation / Income; Ethnicity; Geographical location; Insurance status; Maternal age; Maternal education; Single / Multiple Pregnancy | 2004-2013 | Various – divided into Critical and Noncritical | 15533 | 1289 | Neonates | First year of life | Moderate |
| **52** | Odim | 2006a | The Annals of Thoracic Surgery | USA | N/A | Ethnicity | 1982-2001 | PA+IVS | 106 | 16 (12 of these deaths occurred in first year of life, but only possible to discern race/ ethnicity in 9 of these) | <1 year | Following initial palliation procedure but before definitive surgical repair. | Serious |
| **53** | Odim | 2006b | European Journal of Cardio-Thoracic Surgery | USA | N/A | Ethnicity | 1982-2001 | Biventricular repair of PA+IVS | 56 | 4 | Unclear. Age range of patients who died = 7 days – 17 years | “early death” – all occurred within 25 days of repair | Serious |
| **54** | Taylor | 2016 | Pediatric Cardiology | USA | N/A | Deprivation / Income; Ethnicity; Geographical location; Maternal age; Number of siblings; Parental occupation; Public assistance status; Single / Multiple adult carers | 2000-2009 | HLHS or other related single right ventricle malformations | 273 | 32 | Neonates | Interstage mortality after Norwood procedure | Moderate |
| **55** | Miller | 2010 | The Journal of Pediatrics | USA | Metropolitan Atlanta Congenital Defects Program (MACDP) | Deprivation / Income; Ethnicity; Maternal age | 1979-2003 | AVSD | 338 | 111 | Neonates | Up to 24 years | Moderate |
| **56** | Shin† | 2007 | Birth Defects Research (Part A) | USA | MACDP | Ethnicity | 1979-2003 | Various types of CHD in patients with Trisomy 21. | 334 | 33 | Neonates | First year of life. Mortality further divided into neonatal and post-neonatal periods. | Serious |
| **57** | Siffel | 2015 | Pediatrics | USA | MACDP | Deprivation / Income; Ethnicity; Maternal age; Single / Multiple Pregnancy | 1979-2005 | HLHS | 212 | 160 | Neonates | Up to 30 years follow-up | Serious |
| **58** | Tumin | 2017 | Congenital Heart Disease | USA | United Network for Organ Sharing | Insurance status | 2004-2015 | Heart transplantation in adults with CHD | 470 | 69 | Adults | Mortality prior to returning to work after transplant | Critical |
| **59** | Verheugt | 2010 | European Heart Journal | Netherlands | CONCOR Dutch National Registry | Ethnicity | 2001-2009 | Various | 6933 | 197 | >18 years | Adult mortality | Serious |
| **60** | Wang | 2013 | The Journal of Pediatrics | USA | New York State Congenital Malformations Registry | Ethnicity; Maternal age; Maternal education | 1983-2008 | CoA, HLHS, TGA, TOF | 8181 | 2473 | <=2 years | Over entire study period. Follow-up between 2-25 years. | Serious |
| **61** | Xiang | 2018 | Lancet Child & Adolescent Health | China | N/A | Deprivation / Income | 2012-2015 | Various “complex” CHD | 2555 | 70 | Median age at operation 0.88 years | Crude data only provided for Inpatient mortality; other study analyses focus on post-discharge mortality | Serious |
| **62** | Pasquali | 2012 | Pediatrics | USA | STS-CHSD | Case volume | 2006-2009 | Various | 35776 | 1383 | <18 years (median 6.4 months) | Inpatient mortality | Serious |
| **63** | Welke | 2009 | Congenital Heart Disease | USA | STS-CHSD | Case volume | 2002-2006 | Various – divided into “overall”, “low difficulty” and “high difficulty” | 32413 | 1194 | <18 years | Inpatient mortality | Serious |
| **64** | Vinocur | 2013 | Pediatric Cardiology | North America | Pediatric Cardiac Care Consortium | Case volume | 1982-2007 | Various | 85023 | 5237 | <18 years | Perioperative mortality | Serious |
| **65** | Gutgesell | 1994 | The American Journal of Cardiology | USA | University Hospital Consortium | Case volume | 1989-1992 | TGA | 330 | 49 | Majority neonates | Perioperative mortality | Critical |

AAO: Aortic Arch Obstruction. AVSA: Aortic Valvle and Supravalvular Anomalies. CoA: Coarctation of the Aorta. ECMO: Extracorporeal Membrane Oxygenation. HLHS: Hypoplastic Left Heart Syndrome. PA+IVS: Pulmonary Atresia with Intact Ventricular Septum. PDA: Patent Ductus Arteriosus. TA: Tricuspid Atresia. TGA: Transposition of the Great Arteries. TOF: Tetralogy of Fallot. VSD: Ventricular Septal Defect.

*Numbers used in analyses may differ depending on appropriate number for a given calculation.

**Number of procedures / admissions in given study (rather than individual children or patients), thus cannot rule out double counting of patients if underwent >1 procedure / admission in study period.

† Mortality figures based on percentages presented in study, therefore potential for some inaccuracy in rounding.

N.B. Studies that are grouped together and shaded identify them as being from the same database and time period and include at least some data on the same types of CHD: shaded studies do not appear in the same analyses as one another, to prevent risk of double counting. Where two studies qualify for same analysis, study with greater number of patients used.
